# Supplementary figures and images for: Early onset adult deafness in the Rhodesian Ridgeback dog is associated with an in-frame deletion in the EPS8L2 gene
Source: PLoS One. 2022 Apr 6;17(4):e0264365. doi: 10.1371/journal.pone.0264365 (PMC8985935; doi:10.1371/journal.pone.0264365)

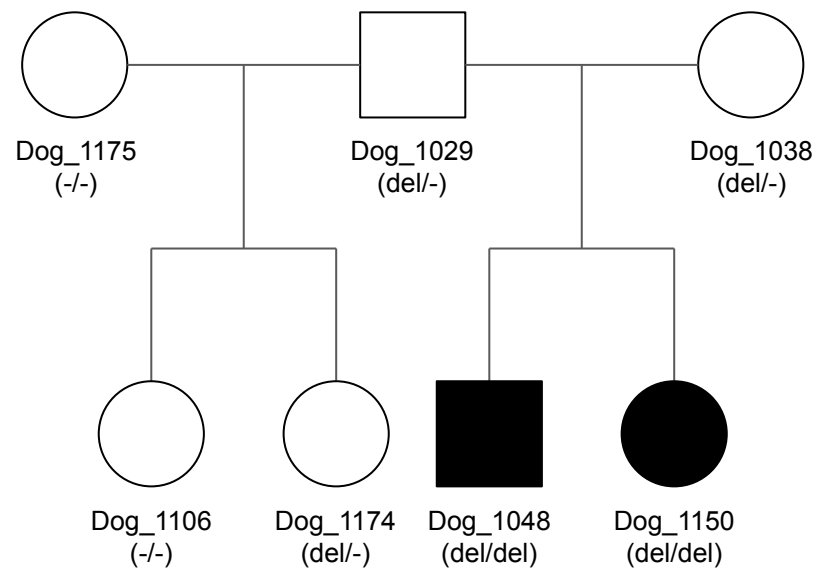

Supplement: S1 Fig — Genotypes of the EOAD-associated 12-bp deletion at CFA18:25868739–25868751 are indicated. (PDF) [file pone.0264365.s001.pdf]

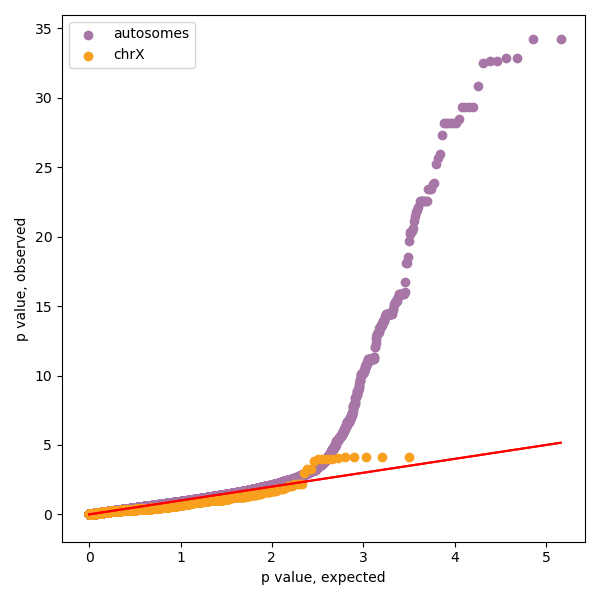

Supplement: S2 Fig — (PNG) [file pone.0264365.s002.png]

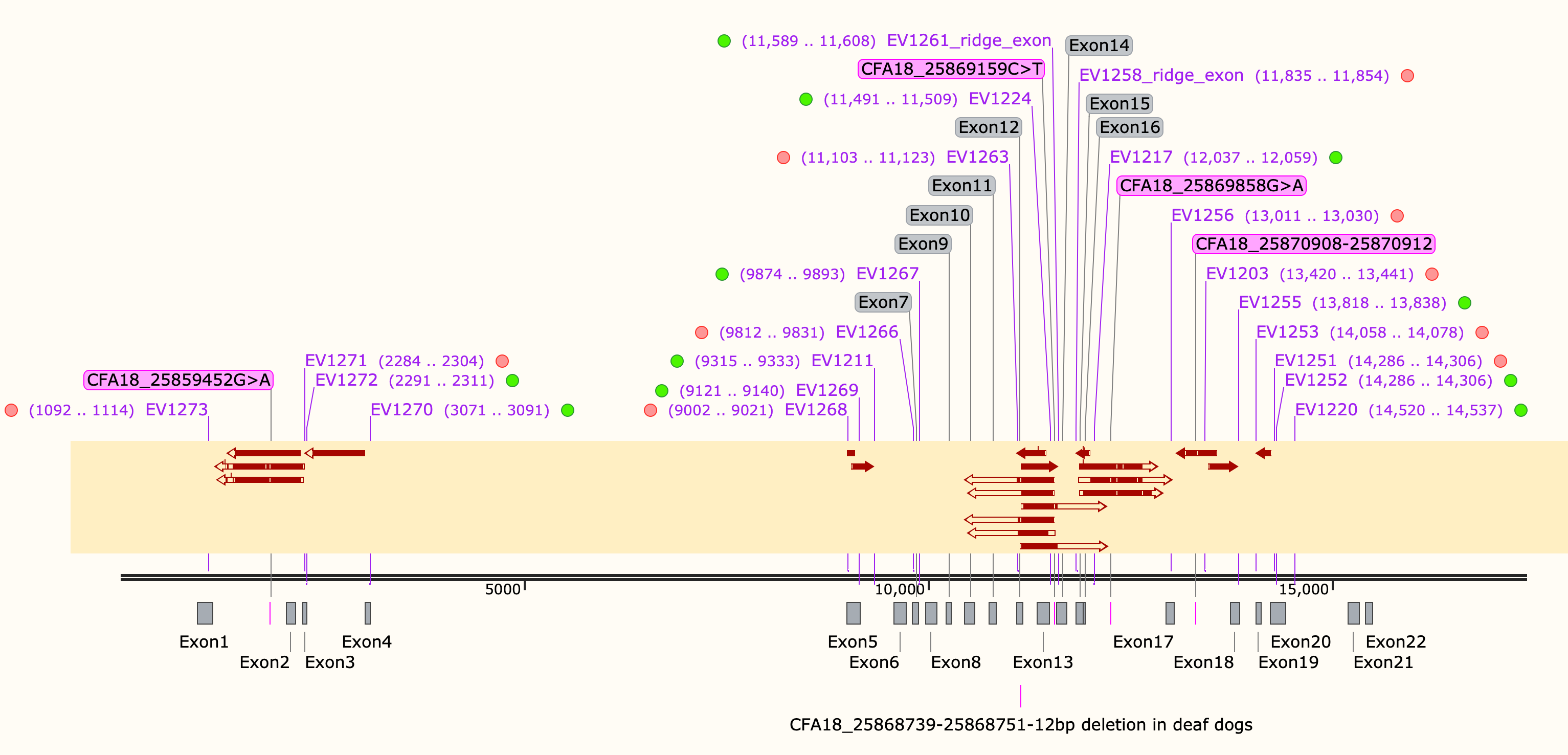

Supplement: S3 Fig — Forward and reverse primers used for PCR amplifications are indicated by red and green circles, respectively. Arrows indicate Sanger sequencing reads of PCR amplicons, where the sequences that are concordant to the reference genome are filled with red, whereas discordant sequences are shown as open symbols, representing SNPs, indels, and low quality sequence regions. The 12-bp deletion associated with EOAD is indicated (CFA18_25868739-25868751-12bp deletion in deaf dogs). (PNG) [file pone.0264365.s003.png]

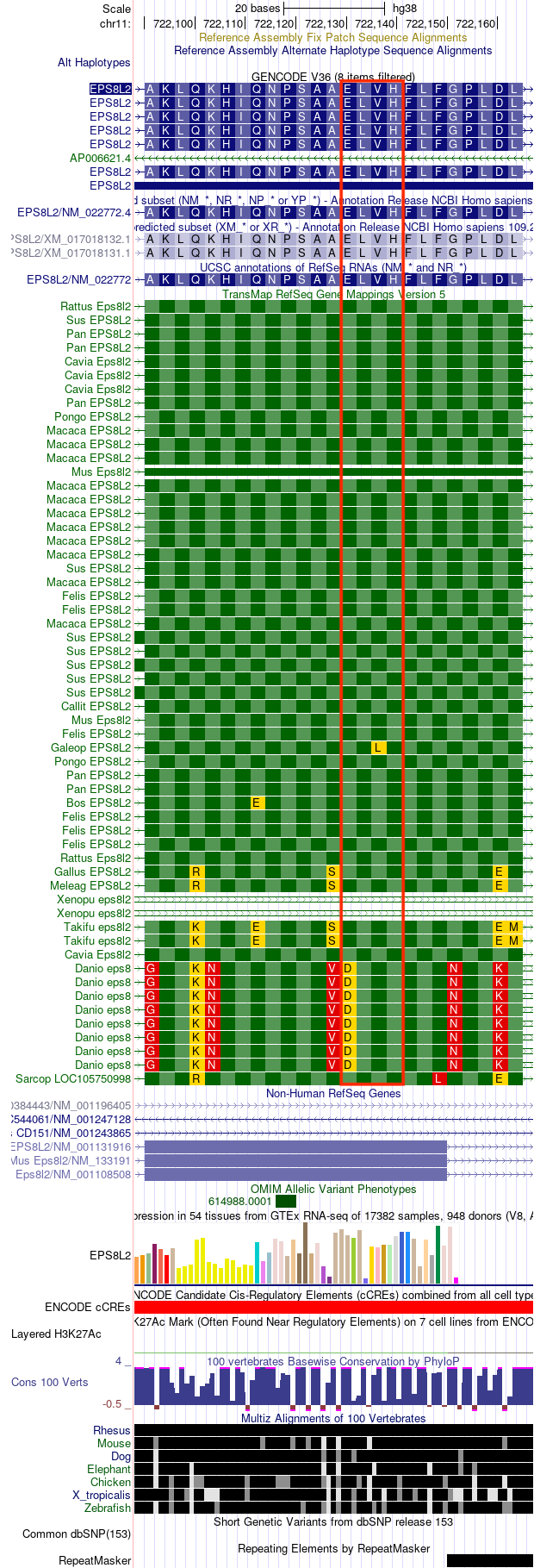

Supplement: S4 Fig — Predicted translated amino acid sequences are indicated in blue with single-letter amino acid codes. Predicted amino acid sequences in other genera/species are indicated in green (amino acids identical to human), yellow (conserved amino acid substitution), and red (non-conserved amino acid substitution). A red rectangle highlights the four amino acid deletion identified in EOAD-affected Rhodesian Ridgebacks in this study. OMIM Allelic Variant Phenotypes (614988.0001) is associated childhood hearing loss reported in Dahmani et al. (2015). (PNG) [file pone.0264365.s004.png]

A)

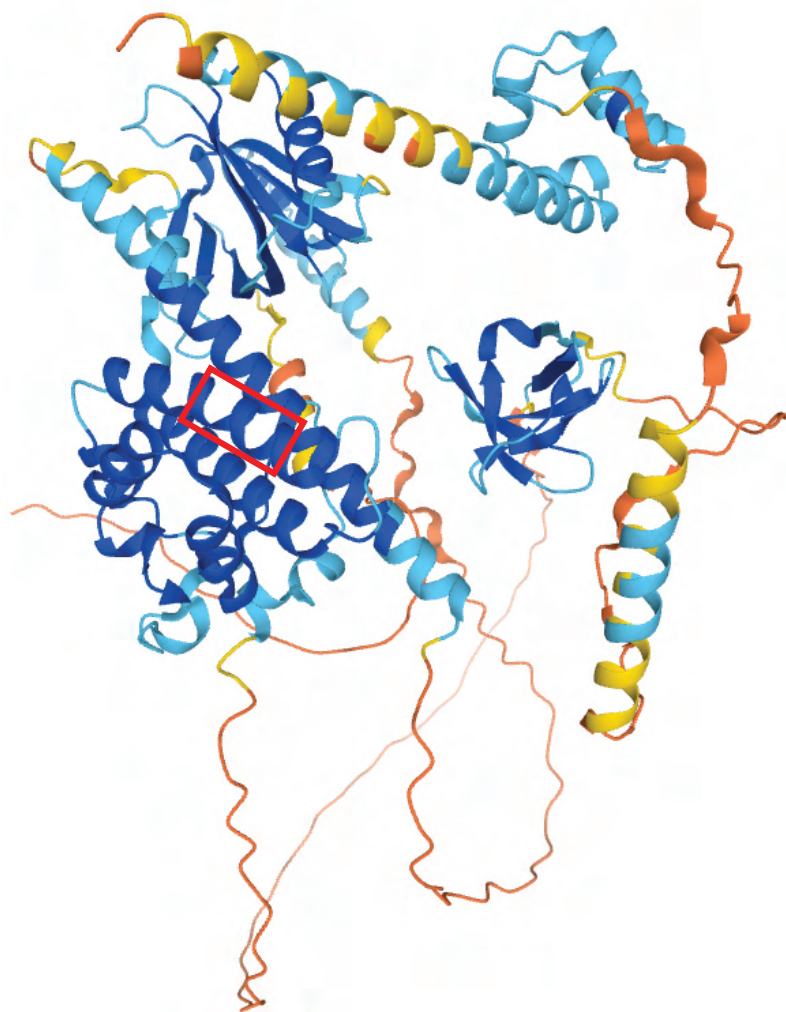

B)

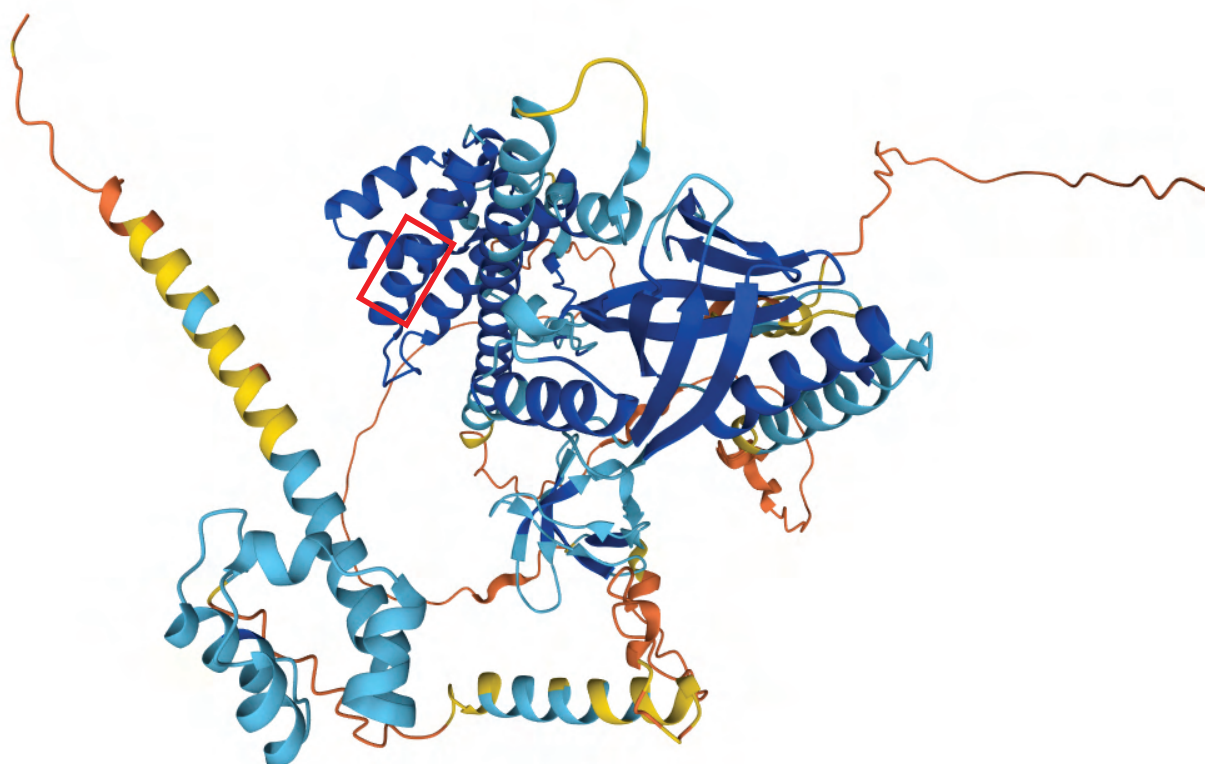

Supplement: S6 Fig — A) Human EPS8L2 (UniPlot ID: Q9H6S3). B) Mouse EPS8L2 (UniPlot ID: Q99K30). (PDF) [file pone.0264365.s006.pdf]
