# Supplementary material for: Early onset adult deafness in the Rhodesian Ridgeback dog is associated with an in-frame deletion in the EPS8L2 gene
Source: PLoS One. 2022 Apr 6;17(4):e0264365. doi: 10.1371/journal.pone.0264365 (PMC8985935; doi:10.1371/journal.pone.0264365)
Supplement: S3 Table — The coordinates of the priming positions are UMICH_Zoey_3.1/canFam5. (PDF) [file pone.0264365.s009.pdf]

S3 Table. A list of primer sequences used for PCR assays and Sanger sequencing analysis. The coordinates of the priming positions are UMICH_Zoey_3.1/canFam5.

A) Primer names and seequences

**Primer name**	**Sequence (5’- 3’)**	**Forward/ Reverse**	**DNA priming region on CFA18**
EV1200	gtttccattcccggctgacg	F	25844765-25844746
EV1201	cccaccttgctgctcctg	R	25842175-25842158
EV1202	agggagcattctgggctcg	R	25843662-25843644
EV1203	CCCTTGAGACACCGTCTGTTCG	F	25844176-25844155
EV1204	aaaatggccacagtgaaacc	F	25856712-25856693
EV1205	gagtgagccgagtgtttgc	R	25856394-25856376
EV1206	gtctgggctgggaggaag	F	25855637-25855620
EV1207	cccatccttgagcctcact	R	25855275-25855257
EV1208	gagctgctcagcgtctcg	F	25854833-25854816
EV1209	CCTCCAAAGCCAGATCCTTG	R	25854264-25854245
EV1210	atggggctgtgtttcttcag	F	25848734-25848715
EV1211	gtggaccggtgtgtgttgt	R	25848281-25848263
EV1212	cagggtctaggtctgcgtc	F	25848187-25848169
EV1213	gaggcagagacggtgctag	R	25847400-25847382
EV1214	aggtttgctggtggatggt	F	25847483-25847465
EV1215	gatggtgctggggatctg	R	25846537-25846520
EV1216	CAGATCCCCAGCACCATCC	F	25846537-25846519
EV1217	CCGGTTCTTGTTTGGAAAAATGC	R	25845559-25845537
EV1218	acagggtctggacaggcaag	R	25844450-25844431
EV1219	cccgagcccagaatgctc	F	25843664-25843647
EV1220	ggtccgctcgtcctctgc	R	25843076-25843059
EV1221	gtagggggcggggctact	F	25842533-25842516
EV1222	GAGCTGAAGAAGGTGTGCG	F	25842331-25842313
EV1223	gaatatccactggggtacacatg	R	25841129-25841107
EV1224	ACCGAGGACGAGTCCATGT	R	25846105-25846087
EV1225	gggcctgcatctccaagt	R	25841854-25841837
EV1250	TCAGCTCGTCCTTGTTGAGC	R	25842344-25842325

EV1251	CAGGAGGATCAGCCACATCAG	F	25843310-25843290
EV1252	CTGATGTGGCTGATCCTCCTG	R	25843310-25843290
EV1253	CAGGCCGGTCTGAAATACTGG	F	25843538-25843518
EV1254	GAGGATGACCACCAGTGGTG	F	25843839-25843820
EV1255	GAGTCTCGTCCAGGATGTTGC	R	25843778-25843758
EV1256	CAACGCCAACGAGCTGTCAG	F	25844585-25844566
EV1257	CTCCAGGACCTCGTCCTTGA	R	25844562-25844543
EV1258	atcccagcaaaccggctatc	F	25845761-25845742
EV1259	gaacttacctgtgagcctgcg	R	25845659-25845639
EV1260	atctccagcttTGAGTGGCC	F	25846007-25845988
EV1261	GGCCACTCAaagctggagat	R	25846007-25845988
EV1262	CTGGATGTGCTTCTGCAGCT	R	25846492-25846473
EV1263	AAGCTGCAGAAGCACATCCAG	F	25846493-25846473
EV1264	gCAAATCCTCAACTGTGCCC	F	25847138-25847119
EV1265	TCTTCCCTTTCTTGCGCTGG	R	25847054-25847035
EV1266	CCTGGTGCATGAGGACATCG	F	25847784-25847765
EV1267	cgcctacTTCAGGGTCTGTG	R	25847722-25847703
EV1268	ACCTGGCCACGTTCATCATG	F	25848594-25848575
EV1269	CGCAGCGACTGATCGTTGAC	R	25848475-25848456
EV1270	GTGGTACTGGGAGGTCTCATG	R	25854525-25854505
EV1271	ggtgtgaccaagatgagtgcc	F	25855312-25855292
EV1272	ggtccttggcactcatcttgg	R	25855305-25855285
EV1273	CGTAACCAGTGTGTGTCTACGTG	F	25856504-25856482

B) Primers pairs that were used for sequencing analysis

**Forward primer**	**Reverse Primer**	**Size (bp)**	**Regions fully or partially covered**
EV1273	EV1272	1220	Exon 1, Intron 1, Exon 2, Intron 2, Exon 3
EV1271	EV1270	808	Exon 3, Intron 3, Exon 4
EV1268	EV1269	139	Exon 5
EV1268	EV1211	332	Exon 5, Intron 5

EV1266	EV1267	82	Exon 7
EV1263	EV1224	407	Exon 12, Intron 12, Exon 13
EV1263	EV1261	506	Exon 12, Intron 12, Exon 13, Intron 13, Exon 14
EV1258	EV1217	225	Exon 15, Intron 15, Exon 16, Intron 16
EV1258	EV1257	1219	Exon 15, Intron 15, Exon 16, Intron 16, Exon 17
EV1256	EV1255	828	Exon 17, Intron 17, Exon 18
EV1203	EV1255	419	Intron 17, Exon 18
EV1253	EV1252	249	Exon 19, Intron 19, Exon 20
EV1251	EV1220	252	Exon 20 and Intron 20
