# Supplementary material for: Early onset adult deafness in the Rhodesian Ridgeback dog is associated with an in-frame deletion in the EPS8L2 gene
Source: PLoS One. 2022 Apr 6;17(4):e0264365. doi: 10.1371/journal.pone.0264365 (PMC8985935; doi:10.1371/journal.pone.0264365)
Supplement: S4 Table — (PDF) [file pone.0264365.s010.pdf]

S4 Table. The number of replicated GWAS runs, where a given marker (CFA18:25,448,444, CFA24:42,714,739 and CFA26:23,554,170) is significantly associated with Early Onset Adult Deafness (EOAD) in Rhodesian Ridgebacks.

	CFA18:25,448,444	CFA24:42,714,739	CFA26:23,554,170
The number of runs	100	0	14
