# Supplementary material for: Early onset adult deafness in the Rhodesian Ridgeback dog is associated with an in-frame deletion in the EPS8L2 gene
Source: PLoS One. 2022 Apr 6;17(4):e0264365. doi: 10.1371/journal.pone.0264365 (PMC8985935; doi:10.1371/journal.pone.0264365)
Supplement: S5 Table — The gene annotation is for canFam3.1 (database version 104.31). (PDF) [file pone.0264365.s011.pdf]

S5 Table. Predicted genes by Ensembl within the EOAD-associated region on CFA18. The gene annotation is for canFam3.1 (database version 104.31)

Chromosome	Start	End	Gene_ID	Gene_name
18	25121175	25202140	ENSCAFG00000006087	septin
18	25345364	25354373	ENSCAFG00000045496	unknown
18	25351512	25352892	ENSCAFG00000006415	secretoglobin
18	25354394	25357367	ENSCAFG00000006423	ODF3
18	25360636	25361299	ENSCAFG00000006433	BET1L
18	25362875	25368378	ENSCAFG00000006439	RIC8A
18	25369382	25388118	ENSCAFG00000006450	SIRT3
18	25388229	25401070	ENSCAFG00000006455	PSMD13
18	25401348	25403139	ENSCAFG00000032118	COX8
18	25433698	25440740	ENSCAFG00000006468	NLRP6
18	25443387	25449405	ENSCAFG00000006474	PGGHG
18	25459469	25460872	ENSCAFG00000050027	unknown
18	25461617	25463721	ENSCAFG00000046009	unknown
18	25484785	25485785	ENSCAFG00000006485	IFITM
18	25487470	25488288	ENSCAFG00000048887	unknown
18	25494018	25494958	ENSCAFG00000030793	IFITM
18	25522852	25532074	ENSCAFG00000006492	B4GALNT4
18	25540073	25547667	ENSCAFG00000006497	PKP3
18	25548637	25553923	ENSCAFG00000006505	SIGIRR
18	25556344	25566407	ENSCAFG00000006512	ANO9
18	25583088	25604213	ENSCAFG00000006515	PTDSS2
18	25606029	25614629	ENSCAFG00000024796	RNH1
18	25642844	25644717	ENSCAFG00000031029	HRAS
18	25647882	25664220	ENSCAFG00000006523	LRRC56
18	25663814	25668768	ENSCAFG00000006526	LMNTD2
18	25668457	25672651	ENSCAFG00000006528	RASSF7
18	25680342	25708502	ENSCAFG00000006539	PHRF1
18	25709049	25709404	ENSCAFG00000006557	IRF7
18	25711706	25733069	ENSCAFG00000006569	DEAF1
18	25733090	25755658	ENSCAFG00000025183	EPS8L2
18	25764171	25771979	ENSCAFG00000029403	transaldolase
18	25773736	25777722	ENSCAFG00000006633	GATD1
18	25824877	25899079	ENSCAFG00000006657	ALKBH3
