# Supplementary material for: Early onset adult deafness in the Rhodesian Ridgeback dog is associated with an in-frame deletion in the EPS8L2 gene
Source: PLoS One. 2022 Apr 6;17(4):e0264365. doi: 10.1371/journal.pone.0264365 (PMC8985935; doi:10.1371/journal.pone.0264365)
Supplement: S6 Table — A) CFA18:25859452G>A in the intron 1. B) A 12-bp deletion at CFA18:25868739–25868751 in the exon 12. C) CFA18:25869159C>T in the intron 13. D) CFA18:25869858G>A in the intron 16. E) A 5-bp deletion at CFA18:25870908–25870912 in the intron 17. Genome coordinates are based on UMICH_Zoey_3.1/canFam5. (PDF) [file pone.0264365.s012.pdf]

S6 Table. Genotype frequencies of the markers in *EPS8L2*. A) CFA18:25859452G>A in the intron 1. B) A 12-bp deletion at CFA18:25868739-25868751 in the exon 12. C) CFA18:25869159C>T in the intron 13. D) CFA18:25869858G>A in the intron 16. E) A 5-bp deletion at CFA18:25870908-25870912 in the intron 17. Genome coordinates are based on UMICH_Zoey_3.1/canFam5.

A) CFA18:25859452G>A

	Case	Control
G/G	7	3
G/A	0	6
A/A	0	8
Total	7	17

B) CFA18:25868739-25868751Del

	Case	Control
wt/wt	0	33
wt/del	0	17
del/del	22	0
Total	22	50

C) CFA18:25869159C>T

	Case	Control
C/C	0	12
C/T	0	0
T/T	9	0
Total	9	12

D) CFA18:25869858G>A

	Case	Control
GG	0	11
GA	1	8
AA	9	0
Total	10	19

E) CFA18:25870908-25870912Del

	Case	Control
wt/wt	1	2
wt/del	0	0
del/del	1	0
Total	2	2
